# Oxidative stress and molecular chaperones: a dynamic crosstalk in neurodegenerative disorders

**DOI:** 10.1515/tnsci-2025-0397

**Published:** 2026-06-24

**Authors:** Leila Noori, Yousef Mohamadi, Francesco Cappello, Federica Scalia

**Affiliations:** Department of Biomedicine, Neuroscience and Advanced Diagnostics (BiND), University of Palermo, Palermo, Italy; Department of Anatomy, School of Medicine, Ilam University of Medical Sciences, Ilam, Iran; Department of Medicine and Surgery, Kore University of Enna, Enna EN, FS, Italy

**Keywords:** oxidative stress, molecular chaperones, neurodegeneration, protein misfolding, cellular proteostasis

## Abstract

Neurodegenerative diseases (NDs), such as Alzheimer’s disease, Parkinson’s disease, and Huntington’s disease are discussed as representative examples of conditions in which oxidative stress and proteostasis imbalance contribute to progressive neuronal dysfunction. Oxidative stress, defined as an imbalance between reactive oxygen species (ROS) production and antioxidant defenses, is widely recognized as a key contributor to ND pathogenesis. Excessive ROS disrupt cellular homeostasis and promotes protein misfolding and aggregation, thereby compromising the proteostasis network. In response to this stress, molecular chaperones, including members of heat shock protein (Hsp) family, play a crucial protective role by maintaining protein folding integrity and preventing toxic aggregation. Furthermore, mesenchymal stem cell-derived extracellular vesicles (MSC-EVs) serve as carriers of bioactive molecules, including antioxidants and molecular chaperones, offering a unique mechanism to restore cellular homeostasis. This review explores the interplay between oxidative stress and molecular chaperones in the pathogenesis of NDs and highlights the emerging therapeutic potential of MSC-EVs as modulators of oxidative and proteostatic imbalance.

## Introduction

Reactive oxygen species (ROS) are natural byproducts of normal metabolic processes and external stimuli. At physiological levels, they regulate essential cellular processes such as differentiation, apoptosis and survival [1]. However, when ROS production exceeds antioxidant defenses, oxidative stress (OS) arises which damages cellular macromolecules, particularly proteins, lipids, and DNA, disrupting cellular homeostasis [2]. Chronic OS is a key deriver of neurodegenerative diseases (NDs), where oxidative damage, coupled with other pathological factors such as protein misfolding and neuroinflammation, accelerates neuronal dysfunction and death [3].

Neurons are particularly vulnerable to OS due to their high metabolic demands, limited antioxidant capacity, and ROS-sensitive membrane lipids. This vulnerability contributes to the progressive neuronal loss observed in many NDs. Alzheimer’s (AD), Parkinson’s (PD), and Huntington’s disease (HD) are discussed as representative NDs to illustrate shared mechanisms involving OS, mitochondrial dysfunction, and impaired protein homeostasis rather than as disease-specific mechanistic analyses. Oxidative damage promotes protein misfolding and aggregation triggering reactive astrogliosis and calcium dysregulation, hallmarks of NDs [4].

Molecular chaperones, particularly heat shock proteins (Hsps), represent a crucial defense against OS [5]. These highly conserved proteins assist in the proper folding and maintenance of cellular proteins, thereby preventing misfolding and aggregation [6]. In response to OS, chaperones are upregulated through the activation of heat shock factor 1 (HSF1), although excessive OS can impair HSF1 function [5]. Chaperone deficiency contributes to neuronal proteostasis collapse and accelerates disease progression [7], 8].

Recent advances highlight the therapeutic potential of extracellular vesicles (EVs), particularly mesenchymal stem cell-derived EVs (MSC-EVs), carrying bioactive proteins, RNAs, and lipids with antioxidative and immunomodulatory properties [9]. However, the role of endogenous EVs in NDs remains controversial. They may also propagate pathogenic molecules underscoring their dual role in disease dynamics [3].

This review summarizes the molecular mechanisms linking OS to NDs, emphasizing the role of molecular chaperones in disease progression and the emerging promise of EV-based therapies to alleviate oxidative damage in NDs.

## ROS generation and types

ROS are produced through various endogenous and exogenous pathways including mitochondrial respiration, enzymatic processes, and environmental exposures [10].

Mitochondria is the primary source of ROS in most eukaryotic cells. During electron transport chain (ETC) activity, which takes place in the inner mitochondrial membrane, electrons occasionally leak and react with oxygen to form superoxide anion (O_**2**_^**−**^), which can be further converted into other ROS, such as hydrogen peroxide (H_2_O_2_) and hydroxyl radicals (•OH), under certain conditions. While ROS generation is a normal byproduct of cellular metabolism, excessive electron leakage or ETC dysfunction elevates ROS production [11], 12].

Various enzymes also contribute to ROS generation. NADPH oxidases (NOXs), a family of enzymes, are one of the major sources of ROS generating superoxide (O_2_^−^) especially in immune cells. NOXs are involved in phagocytic killing of pathogens, but excessive or dysregulated NOX activity is linked to OS and various diseases, including NDs [13]. Further, xanthine oxidase (XO) a form of xanthine oxidoreductase (XOR) contributes to ROS production [14]. XO produces superoxide and hydrogen peroxide during hypoxanthine and xanthine oxidation which is particularly relevant in ischemia-reperfusion injury and certain forms of inflammation [15]. Cytochrome P450 (CYP) enzymes produce ROS as byproducts of their catalytic cycles in which CYP-generated ROS either play a role in contributing to or safeguarding against various phenomena [16]. Likewise, in arachidonic acid (AA) pathway, cyclooxygenases (COX) and lipoxygenases (LOX) oxidize AA into eicosanoids while generating ROS that influences cellular signaling and regulates inflammation, immune defense, and various pathological conditions [17], 18]. In addition, exogenous factors such as ultraviolet (UV) light, ionizing radiation, tobacco smoke, and environmental pollutants are significant contributors to ROS production [19]. UV radiation can excite molecules within the skin and lead to the generation of ROS, which contributes to DNA damage and ageing [20]. Air pollutants like ozone (O_3_) and particulate matter can generate ROS in the respiratory tract, exacerbating inflammatory lung diseases [19]. Moreover, smoking introduces a wide array of ROS and free radicals into the body, contributing to oxidative damage and increasing the risk of many different human diseases [21]. A structural summary of the principal endogenous and exogenous sources of ROS is provided in Table 1.

**Table 1:** Major sources of ROS in human physiology/pathology.

Source	Pathway	Primary produced ROS	Main relevance
Endogenous (mitochondrial)	Mitochondrial electron transport chain (ETC)	Superoxide (O_2_^−^), H_2_O_2_, •OH	Normal byproduct of ATP synthesis; excess causes oxidative stress
Endogenous (enzymatic)	NADPH oxidases (NOX family)	Superoxide (O_2_^−^)	Immune defense; linked to inflammation, neurodegeneration
	Xanthine oxidases (XO activity of XOR)	Superoxide, H_2_O_2_	Involved in ischemia-reperfusion injury, inflammation
	Cytochrome P450 (CYP) enzymes	Various ROS	Drug metabolism, can cause or mitigate oxidative damage
	Arachidonic acid pathway (COX/LOX enzymes)	Various ROS	Regulates inflammation, immune response
Exogenous	Ultraviolet (UV) radiation	Various ROS	Skin aging, DNA damage
	Air pollutants	Various ROS	Respiratory inflammation
	Tobacco smoke	Various ROS	Oxidative damage across multiple systems

Given there are several forms of ROS each with distinct reactivity and biological effects.

Superoxide anion (O_2_^−^), formed when molecular oxygen gains a single electron, is the primary ROS generated as a byproduct of aerobic respiration. Although it is relatively short-lived and less reactive compared to other ROS, it still poses a threat to cellular integrity by causing oxidative damage to proteins, lipids, and nucleic acids. Cells rely on the superoxide dismutase (SOD) enzyme to detoxify superoxide into hydrogen peroxide (H_2_O_2_) [22].

The hydrogen peroxide (H_2_O_2_) is a more stable and membrane-permeable ROS compared to superoxide, allowing it to diffuse freely between cellular compartments. At physiological levels, it is often considered a signaling molecule involved in redox regulation, cell proliferation, and immune responses. However, at elevated concentrations, it can become cytotoxic. When accumulated, it reacts with transition metals (e.g., iron (Fe^2+^) and copper (Cu^+^)) through Fenton chemistry, producing highly reactive hydroxyl radicals (•OH), which can cause severe oxidative damage to biomolecules [23].

The hydroxyl radical (•OH) is one of the most reactive and damaging ROS with an extremely short half-life primarily generated through the Fenton reaction. Additionally, they can be formed *via* the Haber-Weiss reaction, where superoxide (O_2_^−^) and hydrogen peroxide interact in the presence of metal ions. Due to their lack of specificity, hydroxyl radicals cause severe OS [24], 25].

The singlet oxygen (^1^O_2_) is an excited, high-energy form of molecular oxygen (O_2_), significantly reactive compared to its ground-state counterpart and typically generated through photochemical reactions. Due to its reactivity, singlet oxygen readily oxidizes lipids, proteins, and nucleic acids [26].

**Figure 1:**
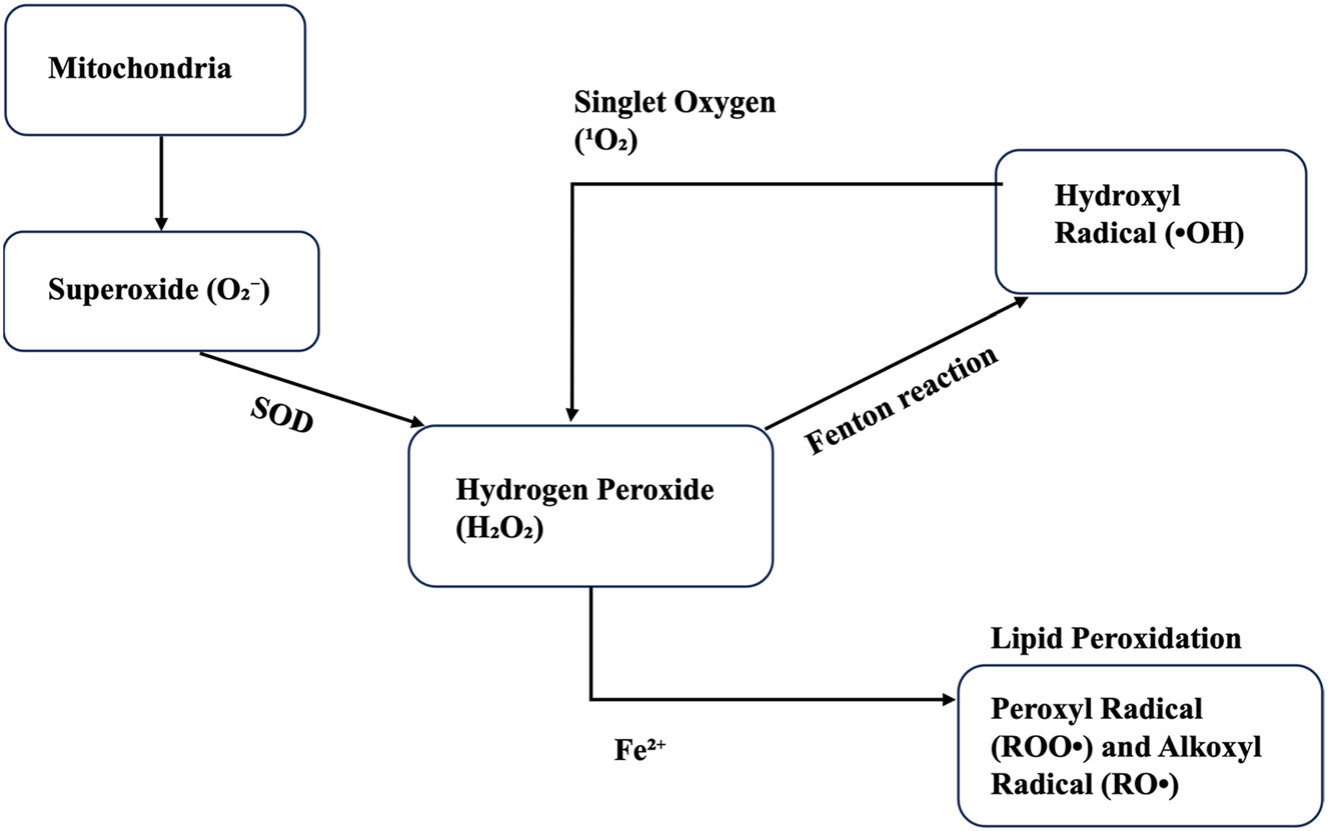
Pathways of ROS generation and interconversion.

The peroxyl (ROO•) and alkoxyl (RO•) radicals are lipid-derived ROS that arise primarily from the oxidation of polyunsaturated fatty acids in cell membranes. These radicals propagate lipid peroxidation that disrupts membrane integrity, alters cellular signaling, and compromises cell function [27]. Figure 1 summarizes the main types of ROS.

### OS and cell response mechanisms

**Figure 2:**
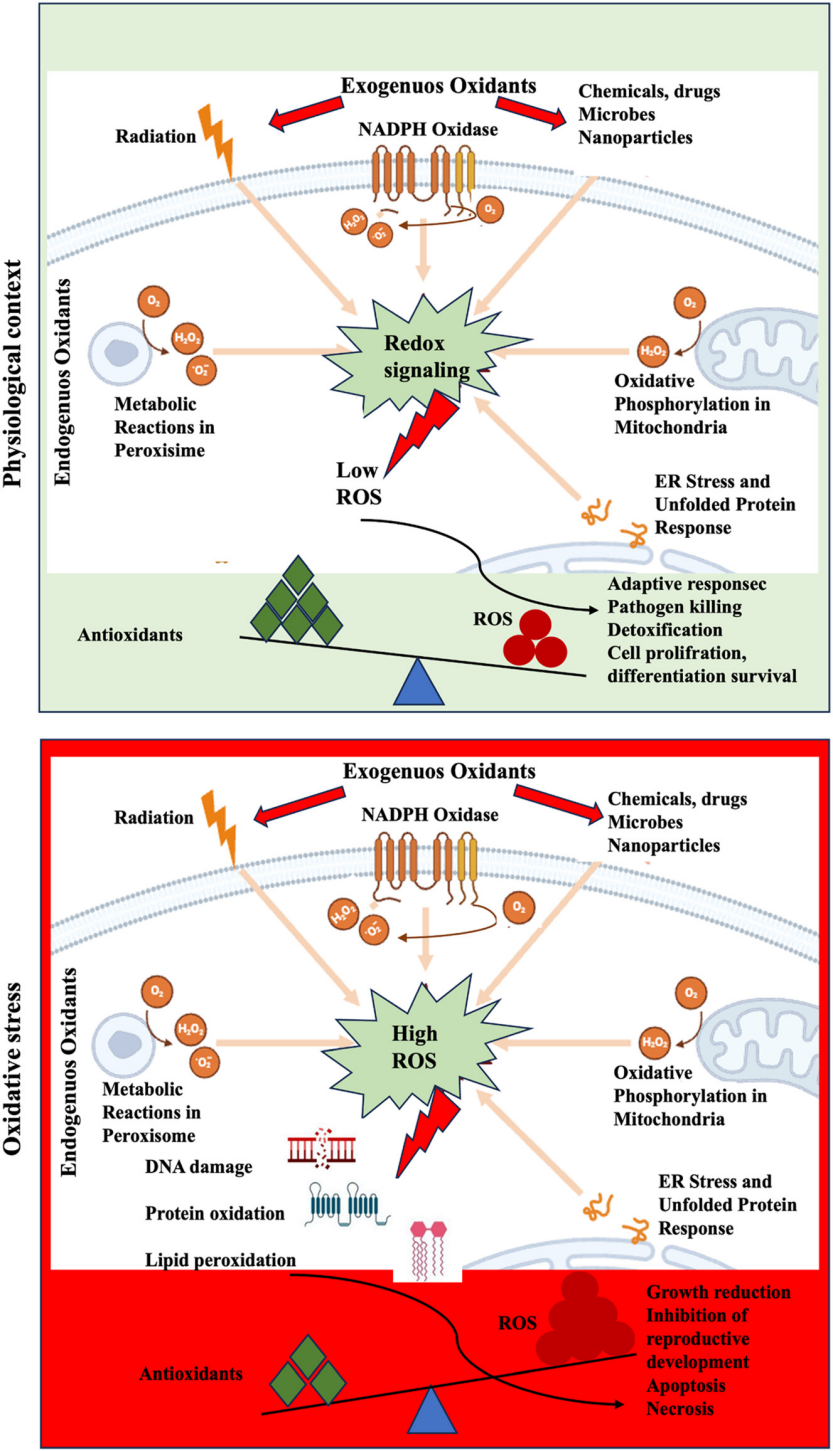
Cellular effects of reactive oxygen species (ROS) under normal and stress conditions. The top panel illustrates physiological conditions where low ROS levels from endogenous and exogenous sources are balanced by antioxidants, supporting redox signaling and adaptive responses such as detoxification, proliferation and survival. The bottom panel shows excessive ROS production overwhelmes antioxidant defenses, leading to oxidative damage on DNA, proteins and lipids, ER stress and adverse outcomes such as apoptosis, necrosis and growth inhibition.

OS refers to a disturbed equilibrium favoring ROS generation over cellular antioxidant defenses (Figure 2). Failing to neutralize ROS, OS ensues and results in molecular damage and impaired cellular function [28]. ROS, particularly hydroxyl radicals, initiate lipid peroxidation, which results in the formation of lipid peroxides. These peroxidized lipids can disrupt cellular membranes, leading to altered membrane fluidity and permeability, and can further propagate cellular damage through the generation of secondary ROS [29]. OS can lead to the oxidation of amino acid residues, particularly cysteine, methionine, and tyrosine. This results in altered protein structure, function, and stability. Oxidized proteins can lose their enzymatic activity or become aggregated, contributing to cellular dysfunction [30]. OS can also cause DNA damage through various mechanisms, including the formation of DNA adducts, single and double-strand breaks, and base modifications such as eight-oxoguanine. Accumulation of oxidative DNA damage can lead to mutations, genomic instability, and contribute to the development of diseases [31].

Cells have evolved various mechanisms to detect and respond to OS. They maintain a sophisticated network of antioxidant enzymes and small molecules to neutralize ROS. Key enzymatic antioxidants include superoxide dismutase (SOD) converting superoxide into hydrogen peroxide, catalase (CAT) detoxifying hydrogen peroxide into water and oxygen, and glutathione peroxidase (GPx) which reduces hydrogen peroxide and organic peroxides. Additionally, non-enzymatic antioxidants such as glutathione, vitamin C, and vitamin E play essential roles in scavenging ROS [32]. Redox signaling pathways are essential for cellular defense against OS, as they regulate processes that determine cell fate. Although excessive ROS levels promote apoptosis, necrosis, and cellular injury, moderate ROS concentrations act as signaling molecules that activate adaptive responses, including autophagy and transcriptional regulation, ultimately contributing to cellular homeostasis. Maitaining a balance between protective redox signaling and oxidative damage is therefore critical for cell survival [33]. Furthermore, the proteostasis network, which includes molecular chaperones, the ubiquitin-proteasome system (UPS), and autophagy, plays a vital role in maintaining protein homeostasis and protecting cells from OS. Autophagy, a key component of this network, functions as a cellular clearance mechanism that degrades and recycles damaged proteins and organelles, preventing their toxic accumulation. Under OS conditions, autophagy is upregulated to remove oxidatively damaged macromolecules, thereby preserving cellular integrity. However, dysfunction in autophagy and proteostasis, such as in Alzheimer’s-like neurodegeneration, leads to the accumulation of misfolded proteins and oxidative damage, exacerbating disease progression. Maintaining a balanced proteostasis-autophagy response is essential for cellular resilience against OS [34].

### The OS contribution in NDs

**Figure 3:**
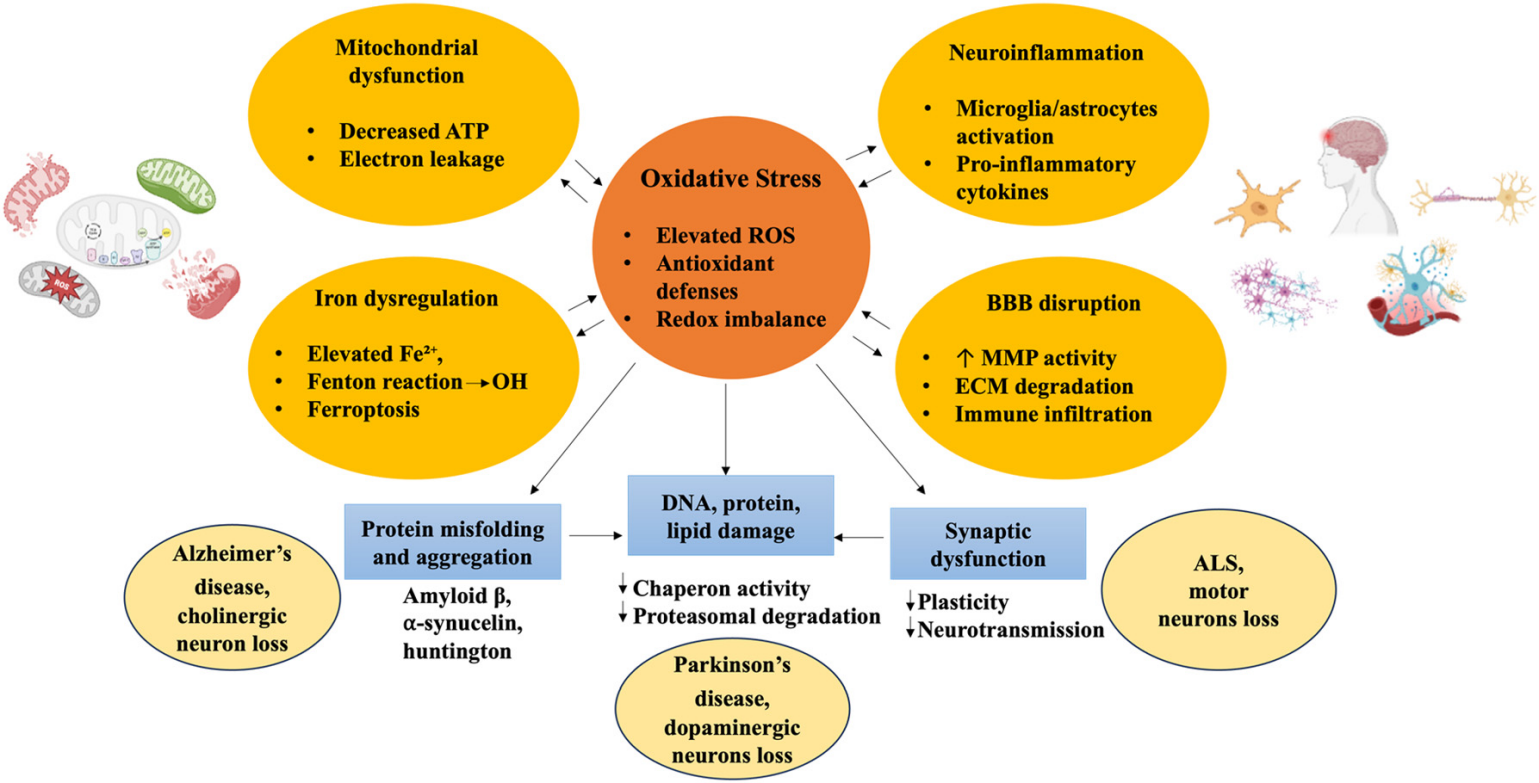
Oxidative stress in neurodegenerative diseases. OS characterized by elevated ROS, impaired antioxidant defenses and redox imbalance arising from mitochondrial dysfunction, iron dysregulation, neuroinflammation and blood-brain barrier disruption that contributes to protein aggregation, macromolecular damage, and synaptic impairment. Ultimately, it can lead to selective neuronal loss in neurodegenerative diseases.

**Figure 4:**
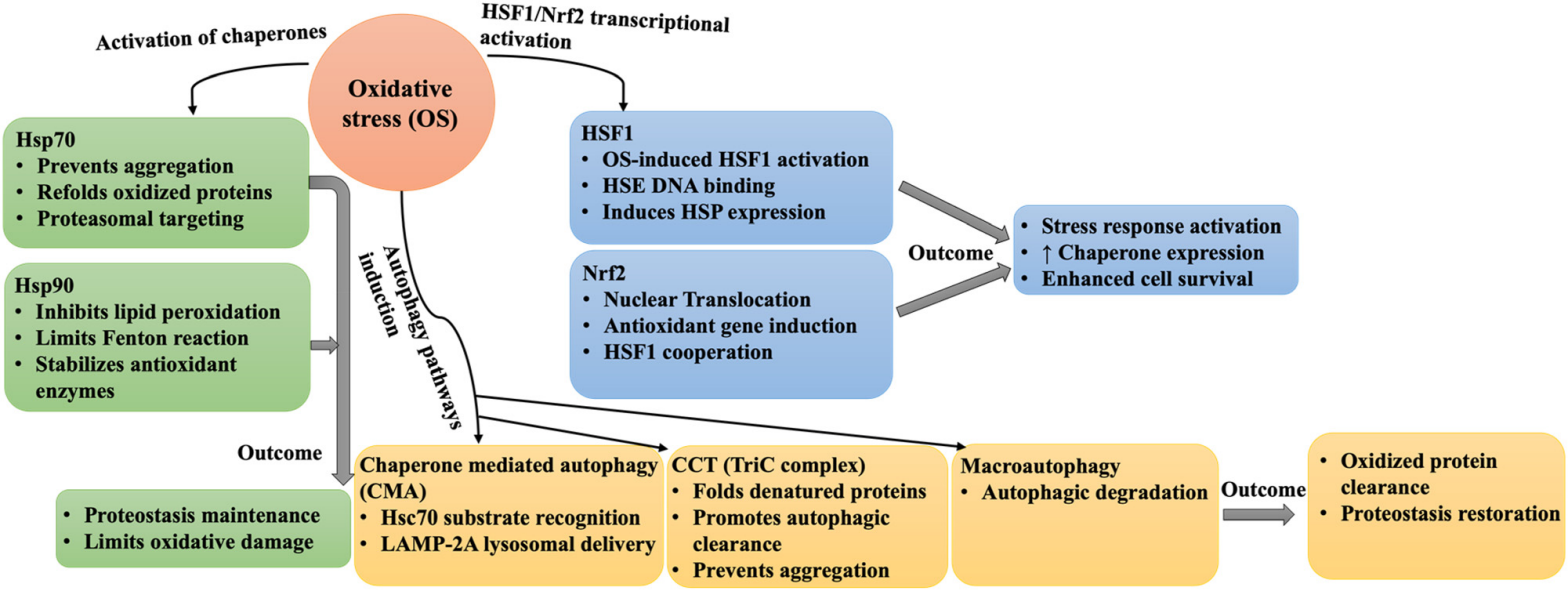
Molecular chaperone response to oxidative stress. OS causes the activation of various defense mechanisms such as molecular chaperones, transcriptional regulators, and autophagy pathways. Chaperones like Hsp70 and Hsp90 prevent protein aggregation, refold oxidized proteins, and inhibit lipid peroxidation. On the other hand, OS activates HSF1 and Nrf2, resulting in increased expression of heat shock proteins and antioxidant enzymes that enhance cellular resistance. Further, OS triggers autophagic mechanisms including chaperone-mediated autophagy (CMA), chaperonin CCT activity, and macroautophagy to selectively degrade damaged proteins and organelles. Consequently, these systems restore proteostasis, reduces oxidative damage, and promotes cell survival.

Persistent OS is implicated in the pathogenesis of numerous diseases including cardiovascular disorders, cancer, age-associated disorders, and neurodegenerative diseases. The central nervous system (CNS) is uniquely susceptible to OS due to its intense metabolic activity, abundance of oxidative-prone lipids and proteins, and relatively limited antioxidant defenses [35]. Neuronal reliance on oxidative phosphorylation generates ROS during aerobic metabolism [36]. While ROS play important roles in cellular signaling, in NDs, sustained ROS accumulation triggers neuronal damage and amplifies neuroinflammation thereby accelerating the progression of disorders like AD, PD, and ALS. Moreover, accumulation of oxidized macromolecules leads to the formation of toxic byproducts that impair cellular function and exacerbate redox imbalance promoting NDs [37], 38]. These alterations further compromise proteostasis, mitochondrial function and synaptic integrity (Figure 3). Understanding these mechanisms highlights the therapeutic relevance of targeting redox imbalance in neuroprotection [39] (Figure 4).

Mitochondria, essential organelles responsible for energy metabolism, lipid biosynthesis, and apoptosis regulation, are at the heart of neurodegenerative pathophysiology. Under normal conditions, mitochondria generate ATP through oxidative phosphorylation, a process that also produces ROS as byproducts [40]. Neurons consume a disproportionate amount of oxygen, about 20 % of the body’s basal intake which makes them especially prone to ROS generation [41]. Mitochondrial dysfunction leads to impaired oxidative phosphorylation, a disruption that affects synaptic plasticity, neurotransmitter production, and overall cellular homeostasis [42]. As mitochondrial function deteriorates, electron leakage from ETC generates excess ROS, further damaging mitochondrial components and amplifying OS. While mild OS can be managed by cellular repair mechanisms like mitophagy (the selective degradation of damaged mitochondria), severe oxidative damage leads to mitochondrial depolarization, the loss of mitochondrial membrane potential, and a breakdown in calcium homeostasis. Mitochondrial damage impairs ATP production and trigger apoptotic pathways and creates a vicious cycle of cellular injury that is central to the pathogenesis of several NDs. Furthermore, mitochondrial dysfunction in NDs is closely linked to calcium dysregulation, another key feature of oxidative damage [43], 44]. This process is especially prominent in diseases like AD and PD where calcium homeostasis is disrupted [38].

Another important feature is selective neuronal vulnerability (SNV), wherein certain neuronal populations are disproportionately affected by OS is a key feature of NDs. Despite the brain’s overall capacity to manage ROS, specific neuronal subtypes such as dopaminergic neurons in the substantia nigra or cholinergic neurons in the hippocampus are more susceptible to oxidative damage [36]. This vulnerability is influenced by several factors, including the neurons’ metabolic demands, their regional differences in antioxidant defenses, and their distinct responses to age-related changes or pathological conditions. Understanding the mechanisms of SNV is crucial for unraveling the complex pathophysiology of NDs, as it helps explain why specific neuronal populations are preferentially targeted in diseases like PD or AD [45]. In addition, iron is an abundant cofactor for enzymes in the cytochrome P450 system, ferritin, and mitochondrial ETC components in the brain. Being crucial for normal neuronal function, its excessive accumulation can exacerbate ROS production through the Fenton reaction, where iron catalyzes the conversion of hydrogen peroxide into highly reactive hydroxyl radicals. Elevated iron levels, combined with existing OS, promote neurotoxicity and neuronal death [46]. Moreover, iron-induced OS is linked to ferroptosis which is characterized by lipid peroxidation and iron-dependent cell death. This mechanism is particularly relevant in NDs, where iron accumulation, oxidative damage, and ferroptotic pathways converge to contribute to neuronal loss [47].

OS also impacts the blood-brain barrier (BBB), which normally protects the CNS from peripheral inflammation and neurotoxic substances. When OS increases, matrix metalloproteinases (MMPs) are activated, leading to the degradation of the extracellular matrix and the disruption of the BBB [48]. This breakdown allows peripheral inflammatory cells, cytokines, and neurotoxic molecules to infiltrate the brain, exacerbating neuroinflammation and increasing the oxidative burden on neurons. Neuroinflammation further amplifies OS in the brain, creating a feedback loop that accelerates neuronal dysfunction and contributes to disease progression. Inflammation-induced OS is particularly damaging because it not only promotes ROS production but also leads to mitochondrial dysfunction, protein aggregation, and cellular apoptosis [49].

Accumulation of misfolded proteins and aggregates such as amyloid plaques in AD, alpha-synuclein aggregates in PD, and huntingtin inclusions in HD is a hallmark of neurodegeneration due to compromised proteostasis during OS [3]. These aggregates can further disrupt protein interactions, hinder essential cellular dysfunction and activate inflammatory pathways [50]. Although molecular chaperones and the ubiquitin-proteasome typically preserve proteostasis, chronic OS can overwhelm these systems [51], 52]. Consequently, OS is closely linked to disruption of protein homeostasis, as excessive ROS promote protein misfolding and aggregation, while the resulting protein aggregates can further exacerbate OS, ultimately contributing to neuronal dysfunction and neurodegeneration [53].

## Molecular chaperones in response to OS

Cells constantly encounter a variety of acute and chronic stressors that necessitate a delicate balance between survival and apoptosis [54]. Molecular chaperones are central to this balance preserving proteostasis by facilitating proper protein folding, directing the degradation of damaged proteins and orchestrating several protective mechanisms including the ubiquitin-proteasome system and autophagy. Through these pathways, chaperones help cells restore redox equilibrium, mitigate oxidative damage, and enable the repair of misfolded proteins or their eventual elimination when irreparable [55].

Major chaperone systems include the Hsp70 and Hsp90 families. Hsp70 functions as a primary folding chaperone that binds nascent or misfolded polypeptides and prevents aggregation while coordinating protein refolding or degradation through interactions with co-chaperones and the ubiquitin-proteasome system. Hsp90 stabilizes a wide range of signaling proteins and client kinases, thereby influencing cellular stress responses and survival pathways [56], [57], [58], [59]. Small heat shock proteins (sHsps), such as Hsp27, function as ATP-independent holdases that bind partially unfolded proteins and prevent their aggregation, facilitating subsequent clearance through autophagy or proteasomal mechanisms [60]. In addition, mitochondrial chaperonins such as Hsp60/Hsp10 are essential for maintaining mitocondrial protein folding and preserving mitocondrial function during OS [61]. A summary of major chaperone families, their mechanisms in oxidative stress responses and associated NDs is presented in Table 2.

**Table 2:** Major molecular chaperones in oxidative stress response.

Chaperone	Function	Mechanisms of action in oxidative stress response	Associated diseases	References
Hsp70/Hsp90	Promote protein folding, stabilization, maturation, and prevent aggregation; support cellular stress resistance	– Bind to unfolded or misfolded proteins to prevent aggregation – Target oxidized proteins for proteasomal degradation – Collaboratively regulate cellular redox balance	AD, PD, ALS, HD	[56], [57], [58], [59]
Small heat shock proteins (sHsps)	Protect cells by preventing protein aggregation under stress	– Act as holdases, binding damaged proteins to prevent irreversible aggregation – Enhance protein clearance *via* autophagy or proteasomal pathways	AD, HD, PD, ALS	[60]
BiP/Grp78 Grp94	ER-resident chaperones critical for protein quality control during the unfolded protein response (UPR)	– Recognize and refold oxidative stress-induced misfolded proteins – Direct irreversibly damaged proteins to degradation pathways	Neurodegenerative and ER stress-related diseases	[79]
Hsc70	Maintain proteostasis by facilitating protein folding and selective protein degradation	– Mediate chaperone-assisted autophagy (CMA) by transporting damaged proteins to lysosomes – Promote selective removal of oxidized proteins during stress	AD, PD	[80]
Hsp60/Hsp10	Ensure mitochondrial protein folding and maintain mitochondrial function under stress	– Assist folding of mitochondrial enzymes – Critical for the folding and function of manganese superoxide dismutase (MnSOD), a key antioxidant enzyme	AD and PD associated with mitochondrial oxidative stress	[61]
CCT (chaperonin containing TCP1)	Support proper folding of newly synthesized cytosolic proteins and prevent aggregation	– Enhance neuronal survival by interacting with oxidatively damaged proteins – Protect against ischemic and oxidative stresses	Neurodegenerative diseases, motor neuron ischemic injury	[81]

OS induces significant post-translational modifications in proteins such as carbonylation and oxidation of amnio acid side chains, which compromise protein structure and function, predisposing them to misfolding and aggregation. In response to such proteotoxic stress, molecular chaperones are strongly upregulated to protect cells from the accumulation of damaged proteins [62]. In eukaryotes, heat shock factor 1 (HSF1) serves as the central regulator of Hsps expression upon OS. ROS can directly induce HSF1 homomultimerization and nuclear translocation or indirectly activate nuclear factor erythroid 2-related factor 2 (Nrf2), which cooperates with HSF1 to enhance antioxidant defenses. The coordinated action of HSF1 and Nrf2 not only supports the cellular antioxidant system but also reinforces the cellular proteostasis network, ensuring proper folding and repair of damaged proteins. OS can influence chaperone activity through redox-dependent modifications that alter their folding capacity and substrate interactions, further highlighting the tight regulatory link between OS signaling and proteostasis maintenance [63], 64].

Among the most studied molecular chaperones, Hsp70 and Hsp90 play pivotal roles in maintaining cellular proteostasis under OS [62]. Hsp70 prevents the aggregation of oxidized proteins and facilitating their degradation through the proteasome. Under oxidative conditions, it collaborates with proteasome to enhance the clearance of oxidized proteins, thereby reducing cytotoxic burden of misfolded proteins and ROS [65], 66].

Similarly, Hsp90, another essential chaperone, protects cells from oxidative damage by inhibiting lipid peroxidation thereby reducing secondary ROS production and their harmful effects [67]. Moreover, Hsp90 acts as an iron-binding chaperone, modulating iron levels to prevent oxidative injury triggered by excess free iron [68]. Notably, Hsp90 interacts with superoxide dismutase 1 (SOD1), a critical antioxidant enzyme whose aggregation is associated with several NDs, regulating its activity and contributing to redox balance [69], 70].

Further, iron dysregulation is a key player in ROS generation and OS [71], and Hsp90 mitigates this OS by inhibiting iron-mediated Fenton reactions thereby minimizing ROS-induced cellular damage including HeLa cells [68].

Molecular chaperones are also involved in selective protein degradation pathways, including chaperone-mediated autophagy (CMA). In CMA, chaperones like Hsc70 (heat shock cognate 70), recognize target proteins and facilitate their transport to the lysosome for degradation providing a more selective mechanism to remove damaged proteins compared to bulk autophagy [72]. ROS-induced knockdown of LAMP-2A, a lysosome-associated membrane protein involved in CMA, impairs the clearance of damaged proteins and promotes caspase-dependent cell death highlighting CMA’s importance in cellular protection (Figure 3) [73].

Molecular chaperones are also central to the unfolded protein response (UPR), activated by accumulation of misfolded proteins in the endoplasmic reticulum (ER) [74]. Under OS, ER-resident chaperones like BiP (Binding Immunoglobulin Protein) and Grp94 are upregulated as part of the UPR. BiP, in particular, is involved in both protein refolding and the degradation of misfolded proteins through autophagy or the proteasomal pathway. OS can reversibly modify BiP’s ATP binding site, reducing ATPase activity and locking it into a holdase state to prevent further aggregation. Once cellular stress is alleviated, co-chaperones like Sil1 restore ATP-dependent activity [75], 76].

Chaperonin CCT, also called chaperonin-containing TCP-1 complex (CCT/TriC), showed a protective role in NDs, inhibiting the aggregation of pathogenic proteins such as mutant huntingtin and tau and enhancing autophagic clearance mechanisms [77]. Impaired CCT function causes accumulation of these toxic proteins, disturbed proteostasis, and neurotoxicity [78]. Notably, mutations in the gene coding for the number 5 subunit of CCT complex have been associated with rare neurodegenerative disorders [6], 52]. Given that beyond folding proteins, CCT modulates autophagy, serving as a crucial defense against protein aggregation in NDs [78].

Moreover, CCT binds HSF1 under physiological conditions keeping it inactive, upon proteotoxic stress HSF1 is released to trimerize, bind DNA, and activate chaperone genes linking protein folding capacity to transcriptional control during OS [82].

Thereafter, mitochondrial chaperones such as mtHsp60 and mtHsp70 are critical for maintaining mitochondrial protein homeostasis. mtHsp60, in particular, ensures proper folding of mitochondrial proteins and prevents aggregation [83]. Elevated mtHsp60 levels in the plasma and brain of AD patients suggest a neuroprotective role, counteracting oxidative damage, reducing amyloid aggregation, inhibiting apoptosis and supporting its potential as a therapeutic target to restore mitochondrial function [84], 85]. However, under severe OS oxidatively damaged mtHsp60 may lose functionality and paradoxically contribute to toxic aggregate formation. Conversely, overexpression of mtHsp60 protects neurons from oxidative damage by restoring mitochondrial function, stimulating its biogenesis and mitigating the pathogenesis of AD and other NDs [86]. mtHsp60 prevents amyloid beta aggregation since its deficiency in neurodegenerative models leads to disease progression [87]. In AD rat models, increased mtHsp60 levels in Purkinje cells of the cerebellum preserve neuronal integrity under oxidative conditions, underscoring its potential as a neuroprotective agent [88], 89].

Another critical mitochondrial chaperone, mtHsp70, is implicated in NDs linked to proteinopathies such as AD and PD. Abundantly expressed in dorsal root ganglion (DRG) neurons, mtHsp70 plays a crucial role in managing OS [90]. Knockdown of mtHsp70 in hippocampal neurons increases resilience to OS-induced cell death, while its overexpression heightens oxidative sensitivity indicating a complex role in mitochondrial proteostasis and neuronal survival [83], 91]. Together, mtHsp60 and mtHsp70 regulate cytotoxic protein aggregates such as amyloid beta and alpha-synuclein, by facilitating protein degradation and enhancing mitochondrial function, thereby maintaining cellular homeostasis. Their interactions, which occur in proximity to mitochondria in neurons and glia, highlight the crucial role of mitochondrial damage in the development of proteinopathies [92]. Age-related oxidative modifications impair these chaperones, contributing to mitochondrial dysfunction, increased OS and progressive NDs [83].

In nervous system glial cells also play a crucial role in modulating neuroinflammation and protecting neurons from oxidative damage. Molecular chaperones such as Hsp27, Hsp47, and HO-1 are upregulated in glial cells in response to OS and participate in maintaining cellular integrity [93], 94]. Hsp27 is primarily produced by astrocytes, stabilizes cytoskeletal proteins such as actin, intermediate filaments, and glial fibrillary acidic protein (GFAP) and prevent oxidative-induced cell death [95], 96]. Similarly, Hsp47 is expressed in glial cells following an excitotoxic injury, supporting ER-related protein folding and stress responses that contribute to neuroprotection and tissue repair [97].

The heme oxygenase-1 (HO-1) pathway also contributes to redox regulation. Induced by ROS, HO-1 converts heme into biliverdin, carbon dioxide, and ferrous iron, thereby reducing oxidative damage [98]. HO-1 plays a dual role, astrocytic HO-1 overexpression offers neuroprotection by reducing heme-induced OS. Although, in AD, early HO activity is linked to OS that suggests a complex role in both neurodegeneration and cellular defense mechanisms [99]. Indeed, HO-1’s antioxidant capacity counteracts OS and potentially slowing down NDs [100]. While HO-1 excessive or prolonged activation may promote iron accumulation and exacerbate neurodegeneration [101]. Further, activation of Nrf2, a stress-responsive transcription factor, upregulates HO-1 expression reducing hydrogen peroxide-induced cell death and offereing further neuroprotection [102].

Given the central role of OS and proteostasis imbalance in NDs, increasing attention has focused on therapeutic strategies capable of simultaneously modulated these interconnected pathways. Among these approaches, MSC-EVS have emerged as promising candidates.

## Antioxidant potential of EVs in OS and NDs: a promising avenue for cell-free therapy

EVs have emerged as promising therapeutic tools for NDs due to their ability to modulate OS, a key contributor to the pathogenesis of disorders such as AD, PD, and HD. EVs are lipid-bilayer nanoparticles released from all cell types and play a fundamental role in intercellular communication by transferring bioactive molecules between cells. Their intrinsic stability, ability to cross the blood-brain barrier (BBB), and capacity to transport diverse molecular cargo make them attractive candidates for cell-free neuroprotective therapies and potential biomarkers for early ND detection [103].

MSC-EVs are particularly enriched in bioactive cargo that contributes to neuroprotection and redox regulation. Their molecular content includes proteins, lipids, and microRNAs that collectively support cellular homeostasis [104]. Proteomic analyses have identified antioxidant enzymes cargo of EVs such as superoxide dismutase, catalase, and glutathione peroxidase, which contribute to the neutralization of ROS and restoration of cellular redox balance [105], 106]. In addition, MSC-EVs contain molecular chaperone including Hsp70, Hsp90, and Hsp27, which are known to support proteostasis by preventing the aggregation of misfolded proteins and facilitating protein quality control mechanisms [107]. Regulatory microRNAs present in EV cargo, including miR-21, miR-124, miR-133b, and miR14a, further modulate pathways associated with neuronal survival, neuroinflammation, and synaptic plasticity, thereby contributing to neuroprotective signaling in recipient cells [108].

Following their release, EVs interact with target cells through several uptake mechanisms, including endocytosis, macropinocytosis, phagocytosis, or direct membrane fusion, enabling efficient delivery of functional cargo to recipient cells. Once internalized, EV components modulate multiple intracellular signaling pathways associated with neuroprotection and cellular stress responses [104]. Among these, activation of PI3K/Akt and MAPK/ERK signaling contributes to the attenuation of neuroinflammatory responses. Importantly, EV cargo can also activate the Nrf2 antioxidant pathway, a central regulator of cellular defense against OS, thereby enhancing endogenes antioxidant capacity and maintaining redox homeostasis in recipient cells [109].

Accumulating evidence from *in vivo* models of NDs supports the therapeutic potential of MSC-EVs. In experimental models of AD, EV administration has been shown to reduce amyloid-beta accumulation and mitigate neuroinflammation [110], while in PD models MSC-EVs have demonstrated protective effects on dopaminergic neurons and improved behavioral outcomes [111]. Similarly, EV-mediated delivery of molecular chaperones and antioxidant enzymes has been associated with enhanced neuronal recovery in models of seizure-induced hippocampal injury through activation of the Nrf2 signaling pathway and reduction of oxidative damage [9], 105]. Additional studies have shown that chaperone-enriched EVs, particularly those containing Hsp27, contribute to cellular protection, maintenance of protein homeostasis, and modulation of immune responses [112]. Moreover, chaperone-associated pathways involving Hsp60 have been linked to improved neuronal survival and behavioral recovery in PD models following transplantation of human umbilical cord mesenchymal stem cell derived dopaminergic-like neurons [113]. The neuroprotective of Hsp70 and Hsp90 further extend to prevention of toxic protein aggregation, including interactions with amyloid-β in AD and α-synuclein in PD, thereby limiting proteotoxic stress [114].

Collectively these findings highlight the capacity of MSC-EVs to simultaneously regulate OS, proteostasis, and neuroinflammatory pathways, positioning them as a promising cell-free therapeutic strategy for NDs. By delivering their unique cargo, EVs can target multiple pathogenic processes associated with neuronal degeneration. Consequently, EV-based and nanomaterial-mediated antioxidant delivery systems represent an emerging avenue for mitigating OS, improving mitocondrial function, and slowing the neurodegeneration processes [3], 115].

Notably, despite their promissing therapeutic potential, several challenges must be addressed before MSC-EVs can be widely translated into clinical therapies for NDs. One major limitation is the intrinsic heterogeneity of EV populations. EVs consisto f diverse subtypes whose composition varies depending on the cellular source, donor characteristics, culture conditions, and isolation techniques. Such variability can significantly influence cargo composition and therapeutic efficacy, highlithing the need for standardized isolation and characterization protocols [104].

Manufacturing and scalability also represent clinical challenges. Clinical application requires large-scale production of EVs under good manufacturing practice (GMP) conditions while maintaining consistent quality and biological activity. Current isolation approaches, including ultracentrifugation, size-exclusion chromatography, and filteration-based techniques, may differ in yield, purity, and reproducibility, complicating the development of standardized production pipelines [116].

Another important issue concerns targeting specificity and biodistribution. Following systemic administration, EVs may accumulate in peripheral organs such as the liver, spleen, and lungs, which can limit their therapeutic delivery to the central nervous system. Although EVs possess the capacity to cross BBB, strategies to enhance targeting efficiency such as surface engineering or ligand modification, are actively being investigated. Finally, regulatory and translational considerations remain significant barriers. The development of EV-based therapeutics requires rigorous characterization of vesicles composition, reproducibility of manufacturing processes, and compliance with regulatory frameworks governing advanced biological products. Addressing these challenges Will be essential for the successful clinical translation of MSC-EV therapies for neurodegenerative disorders [117], 118].

## Conclusions

This review highlights OS as a central deriver in NDs. While ROS are vital for redox signaling and cellular regulation, chronic ROS overproduction disrupts homeostasis, leading to mitochondrial dysfunction, protein misfolding and neuroinflammation in AD, PD and HD [119]. The interplay between oxidative damage, iron dyshomeostasis, BBB disruption, and protein aggregation reinforces the multifactorial nature of neuronal vulnerability and the selective susceptibility of specific neuronal populations to oxidative insults [36], 120]. Despite intrinsic antioxidant and autophagic defenses, these systems often fail under sustained oxidative conditions, particularly in aging neurons [121].

Proteostasis disruption is a critical consequence of OS, wherein molecular chaperones play a central defensive role. These proteins ensure proper protein folding, prevent aggregation, and facilitate the clearance of oxidized proteins *via* proteasomal degradation and autophagic pathways [122]. Chaperones operate in concert with key stress-responsive mechanisms including the heat shock response (HSR), unfolded protein response (UPR), and autophagy, to preserve proteome stability [123], but chronic OS can compromise these defenses, worsening proteotoxic stress [74].

Among heat shock proteins, Hsp70 is markedly upregulated after OS or protein damage where it inhibits apoptosis and supports immune regulation [124]. In AD brains, Hsp70 protects neurons counteracting Aβ accumulation and neurotoxicity [125]. Further, Hsp90 accumulation in amyloid-rich brain regions suggests a role in Aβ clearance, though reduced serum Hsp90 may reflect compromised OS defense [85]. Enhancing chaperone function through pharmacological or genetic strategies may therefore reinforce proteostasis and slow NDs [126].

In this context, EVs, particularly those derived from MSCs, represent a novel approach for combating OS in NDs [127]. MSC-EVs carry molecular chaperones, antioxidants and signaling factors that activate redox-balancing pathways such as Nrf2, promoting protein refolding and neuronal survival [128]. Their stability and ability to cross the blood-brain barrier make them ideal for targeted neuroprotection, overcoming the limitations of conventional antioxidant therapies [129]. Combining EV-based delivery with nanoparticle carrying antioxidant enzymes could further enhance the therapeutic efficacy while minimizing toxicity [130].

MSC-EVs should also be considered within the broader landscape of emerging therapeutic strategies for NDs. Traditional antioxidant therapies, including compounds such as N-acetylcysteine, coenzyme Q10, and mitochondria targeted antioxidants, aim to mitigate OS but have shown variable clinical efficacy, partly due to limited brain delivery and narrow mechanistic targets [131]. Similarly, small-molecule modulators of molecular chaperones and proteostasis pathways, including pharmalogical regulators of Hsps, have been investigated to enhance protein quality control; however, these approaches may suffer from off-target effects and limited pathway specifity [132]. Gene therapy strategies, including viral vector-mediated gene delivery or gene-silencing approaches, hold promise for targeting disease-causing mutations in disorders such as HD or PD, but challenges remain regarding delivery efficiency, safety, and long-term regulation of gene expression [133]. Stem cell transplantation represents another therapeutic avenue aimed at replacing damaged neurons or providing trophic supports; nevertheless, concerns related to cell survival, immune compatibilty, and potential tumorigenicity persist [134]. In this context, MSC-EVs offer a complementary strategy, combining the advantages of cell-free therapy with the ability to deliver complex molecular cargo that can simultaneously modulate multiple pathogenic pathways [135].

Collectively, this work emphasizes OS as a key pathological mechanism in NDs and underscores the therapeutic promise of chaperone-based and EV-mediated strategies aimed at restoring redox homeostasis and neuronal integrity.

## Challenges and future directions

Despite progress in understanding the links between OS, chaperones and NDs, major challenges remain. Specifying therapeutic targets appears difficult due to overlapping redox and chaperone pathways. Predominantly, data emerge from *in vitro* and animal models does not accurately capture the human complexity or chronic progression of NDs. Future research should prioritise integrative approaches combining advanced imaging, patient-derived models and multi-omics profiling to better characterize oxidative and proteostatic dysfunction. Further exploration of MSC-EVs is needed to optimise large-scale synthesis, cargo standardization and effective blood-brain barrier transport. Clinical trials must clarify the molecular mechanisms underlying EV-mediated neuroprotection and chaperone regulation. Finally, interdisciplinary efforts incorporating molecular biology, nanotechnology and neuroscience will be crucial to develop personalized therapies that restore redox balance and proteostasis in NDs.
